# High Pressure Microscopy of the Silver and Cuprous Halides

**DOI:** 10.6028/jres.068A.008

**Published:** 1964-02-01

**Authors:** A. Van Valkenburg

## Abstract

Using a diamond pressure cell and a polarizing microscope, visual observations were made on the transformations of silver and cuprous halides at calculated pressures up to 125 kilobars. A new birefringent phase was observed in silver iodide at 2400 bars. Four phases were observed in CuI and CuBr while CuCl appeared to have only three.

Using a diamond pressure cell previously described [1]^1^ and a polarizing microscope, visual observations were made on the transformations of silver and cuprous halides at calculated pressures up to 125 kilobars. The object of this investigation was to determine the nature and optical characteristics of new phases resulting from polymorphic changes that may occur in any one of these halides. It has been previously demonstrated, that pressures in the diamond cell are greatest at the cell center and least at the edges. This gradient serves a very useful purpose in enabling one to observe several polymorphic phases occurring in the same field of view at the same time, the denser phases always occurring nearest the cell center. Phase changes occurring as a result of applied pressure were detected by observing changes in indices of refraction between adjoining phases using the well-known Becke line movement technique [2]. Under the microscope a thin white pencil line (Becke line) is observed moving towards the denser phase as the viewing tube is raised; the movement is reversed when the tube is lowered.

Phase changes were also observed by detecting birefringence changes between crossed nicols and by noting changes in absorption in both white and monochromatic light. The various optical effects were observed under the microscope at magnifications of 160 and 400 diam. Pressure values as used in this paper will be confined to the bar^2^ which is defined as 10^6^ dynes/cm^2^.

Accurate pressure measurements are difficult to obtain in the diamond cell since friction and flow shear generate pressure gradients across the anvil faces. These gradients on a first compression may be very large especially when the sample is in a powdered form. An explanation for this is that, on the first compression, the movement of material is large compared with the movement for subsequent pressure cyclings. When the cell is newly charged with powdered material, there is a large volume of unoccupied interstitial space. On the first application of pressure, material is squeezed into these spaces and at the same time flows towards the cell edge. It is this movement at the beginning of compression that produces the greatest pressure at the center of the anvil. As compression is increased extrusion along the cell edge is prevented both by the internal friction of the material and the friction between it and the diamond surfaces. On the second and subsequent compressions the internal movement is relatively small, since the material has been previously compacted. Thus the pressure is distributed over a larger area and the buildup of very high pressure at the center is reduced. If pressures are calculated for the cell using the method in which the force is divided by cross-sectional area, one will obtain large errors, especially on the first compression of the material. The explanation for this is that the method assumes that pressure is equally distributed over the anvil surfaces whereas in practice gradients always exist. This point can be illustrated as follows: The maximum pressure calculated under this assumption for one of the diamond cells, was approximately 70 kilobars. A transition that occurs in AgI at 115 kilobars [4] was observed and photographed at a pressure of 60 kilobars calculated in this manner. These calculations were made on the first compression of the sample. On cycling the pressure, the transition did not appear at the maximum pressure of the cell, thus indicating the reduction of the highest pressure in the sample. By cycling the pressure in the cell, pressure measurement errors have been reduced to values of 5 percent or less in the range below 50 kilobars and to 10 percent in the higher pressure range.

## Silver Halides

The silver halides, AgI, AgBr, and AgCl were obtained in powdered form from commercial sources, and samples were used directly from the bottle. When the powdered sample is squeezed, it forms a uniform transparent surface that is ideal for detecting optical changes. The stable forms of silver iodide at room temperature and pressure are the hexagonal or wurtzite type structure and the cubic or sphalerite type structure [5]. A microscopical examination of the sample indicated that both forms were present but it was estimated that the hexagonal form comprised 90 percent of the sample by volume. Both silver bromide and silver cholride have the sodium chloride type structure at room pressure and temperature [6, 7]. The latter two samples contained less than 0.5 percent impurities by volume.

## Silver Iodide

**Figure 1:**
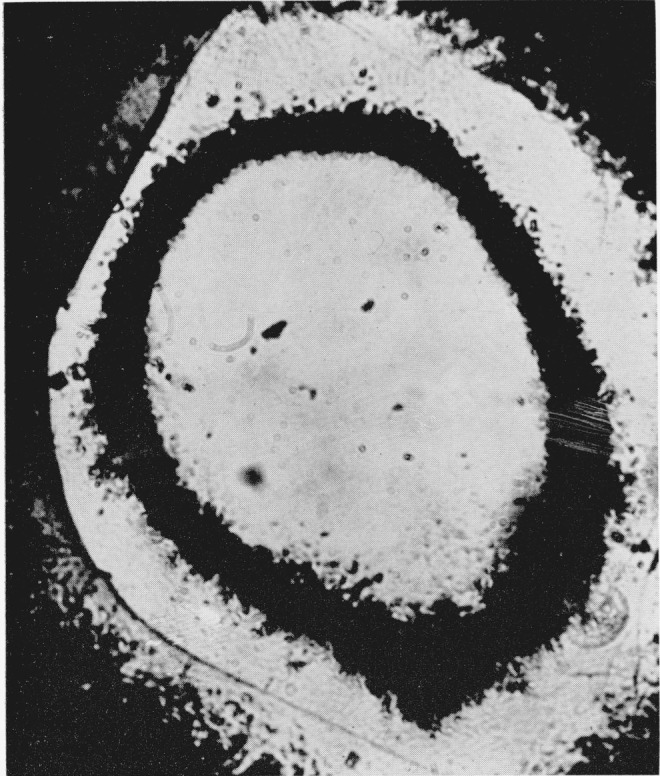
Silver iodide showing the presence of three phases. Pressure 4,000 bars. Magnification 160×.

The hexagonal silver iodide crystals when viewed under the microscope without the application of pressure are lath-shaped with an average grain size of 0.024 mm. The crystals are birefringent when viewed between crossed nicols. As pressure is applied to the cell, they are squeezed together, losing their individual identity. At a calculated pressure of 2400 bars the central portion of the sample begins to darken when observed in white light. With an increase in pressure of approximatley 300 bars the darkened area spreads out from the center. When the dark area is viewed in monochromatic light, using a grating type monochromator with a tungsten light source, it appears opaque. As pressure is increased to 2900 bars the center of the darkened area becomes transparent. In white transmitted light the transparent area has a light amber color and it is optically cubic with a sodium chloride structure [8]. When viewed in monochromatic light this center area begins to absorb strongly at approximately 514 m*μ* and it appears to be completely absorbing at 450 m*μ*. However, on careful examination at 450 m*μ* the area appeared to exhibit a dark reddish color that may be interpreted as a fluorescence phenomenon. Figure 1 shows the three phases of silver iodide photographed at approximately 4000 bars.

**Figure 2:**
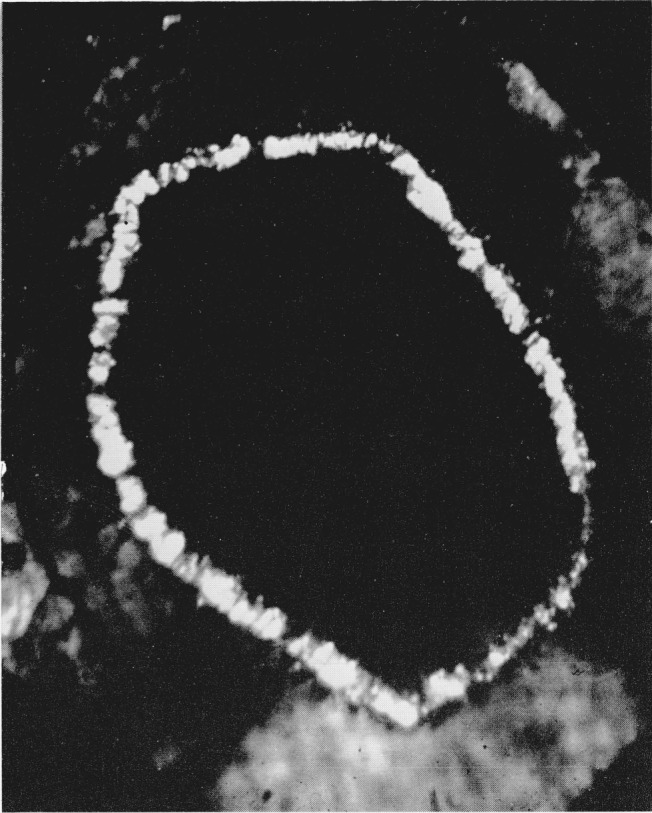
Silver iodide as in figure 1, showing crystallization of dark ring; photographed between crossed nicols. Magnification 160×

After standing for several hours the dark ring of material surrounding the amber area crystallized. When viewed in the polarizing microscope individual crystals measuring up to 100 microns in diameter were strongly briefringent and exhibited bright interference colors, see figure 2. The crystals have positive elongation and their long direction parallels the pressure gradient. In monochromatic light the crystals began to absorb at 473 m*μ* and total absorption occurred at 460 m*μ*. There was no indication of any fluorescence in these crystals. If the diamond cell was jarred or if the pressure was slightly modified the individual crystals readily broke up into smaller domains without any further regrowth. The refractive index of these crystals as detected by the Becke line movement was considerably higher than that of the low pressure phase, but lower than the amber colored area. Using Bridgman’s compressive data to obtain the densities of the silver halides [9], the refractive indices of the polymorphic phases were calculated using Gladstone and Dale’s equation of $\frac{n-1}{d}=K$, where *n* is the mean refractive index, *d*= density and *K*= specific refraction [10]. Table 1 gives the refractive index values of the polymorphic phases and their differences just before and after transition.

**Figure 3:**
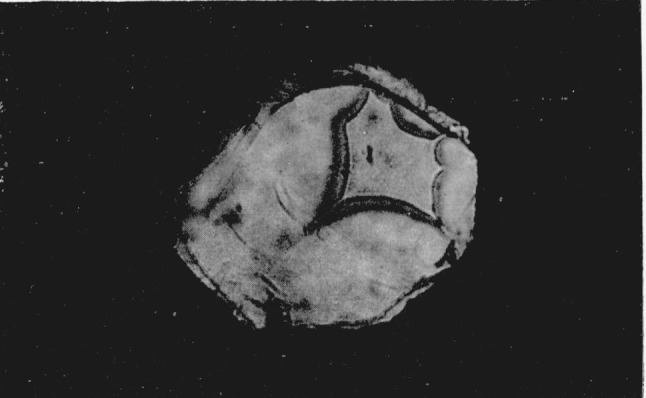
Silver iodide transition at 115 kilobars. Magnification 160×.

**Figure 4:**
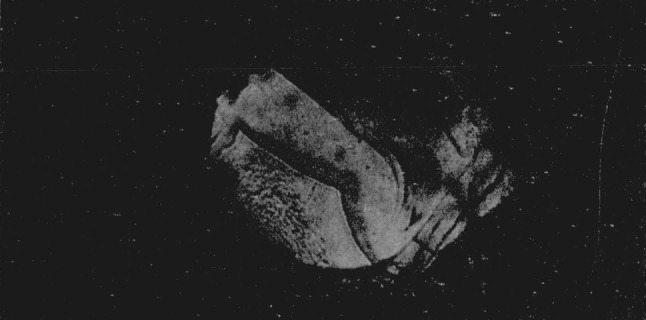
Silver iodide showing ridge like surface. Magnification 160×.

Since index differences >0.003 can be detected microscopically, the boundary between adjacent polymorphic phases can readily be observed in the silver halides. A third transition was observed in AgI using a diamond cell that could attain a calculated pressure of 125 kilobars. At a calculated value of 90 kilobars the sample center began to darken as observed in white light. With the pressure increased to about 100 kilobars a transition occurred with the emergence of a new phase (Drickamer places this transition at about 115 kilobars [4], see fig. 3). (Area in upper right hand section.) The Becke line moved quickly into the new phase when the viewing scope was raised indicating a large difference in the refractive indices of the two phases. It was estimated that the new phase had an index of refraction which was 0.06 greater than the adjacent phase. This would indicate a volume change in the order of 10 to 20 percent. As pressure was increased the central area of the new phase changed in appearance with the formation of a ridgelike surface, see figure 4. (Area in upper right hand section.) This photograph was taken at a later time using a fresh sample. The off-center position of the transition is caused by a misalinement of the diamond anvils, and the area in the lower portion of the photograph shows small fractures in one of the anvils. The average length of the ridges in the high pressure phase is about 10*μ.* Between crossed nicols the area appeared to be birefringent with domains that had an apparent extinction. At these high pressures the diamonds showed strain birefringence but it was possible to distinguish this from the sample birefringence. In general, the strain birefringence of the diamond was wavy in character between crossed nicols while extinction in the birefringent crystals was sharp. There is also the possibility of strain birefringence in the sample itself at these ultra high pressures but this does not appear to be a significant effect. The boundary area between the birefringent phase and the adjacent phase is also well defined indicating the absence of strain. On releasing pressure the birefringent phase disappears; however, the darkened area adjacent to the birefringent phase does not disappear immediately but remains for several hours before it becomes clear and transparent. This absorbing or darkened area shows absorption throughout the visible spectrum. When pressure is totally released the sample transforms back to a cubic or sphalerite form. The hexagonal or wurtzite phase does not reappear.

## Silver Bromide

The sample has a very bright yellow color as taken from the bottle. When pressure was applied to the sample, a transition was observed at approximately 90 kilobars [9] with the familiar appearance of a small island surrounded by the familiar Becke line. When the high pressure phase first emerged the area was clear and transparent and it appeared to be optically isotropic, indicating that the material is cubic. As pressure increases the high pressure phase spreads out and the central area develops a ridgelike surface, similar to that observed in the silver iodide phase at 100 kilobars. This surface when viewed between crossed nicols appears to have a birefringent character. An apparent extinction was observed on a few small grains of about 4*μ* in size at a magnification of 400 diam. Although the evidence is not conclusive, the presence of birefringent particles would indicate that the high pressure phase is anisotropic in character. Bridgman [12] reports transitions in both AgCl and AgBr at 13 kilobars and ambient temperatures using shearing and volume change techniques. A microscopical examination of these two compounds, at pressure ranging from 5 to 20 kilobars did not indicate the presence of any transitions.

## Silver Chloride

The material used in this experiment was obtained from a single crystal of silver chloride of optical grade quality by slicing an area from the crystal that was approximately 2 mm^2^ by 0.5 mm thick. This was then placed between the diamond anvils and squeezed. The transition that occurs near 90 kilobars [9] is rather sluggish as compared with the transition in silver bromide. It was necessary to wait several minutes after a calculated pressure of 100 kilobars had been reached before the formation of the island with the characteristic Becke line was observed. In contrast to the high pressure phase in silver bromide, the high pressure phase of silver chloride, when observed at a magnification of 160 times, was clear and not ridgelike in appearance. Optically the high pressure phase appeared to be cubic and there was no detectable absorption or fluorescence in the visible spectra.

Silver chloride is used in many laboratories as a pressure transmitting medium, especially in the calibration of equipment that uses pyrophyllite or similar compounds for reaction chambers. At low pressures or at pressures under 50 kilobars silver chloride appears to undergo plastic deformation easily. It is interesting to note that the silver chloride transition observed in the diamond cell was obtained without the use of gasket material. There was no tendency for the silver chloride, which had an approximate thickness of 4*μ* to extrude or flow by plastic deformation even after a period of several hours. The friction between the diamond surfaces and the silver chloride, plus the internal friction of the silver chloride, was evidently sufficient to prevent extrusion.

## Cuprous Halides

The cuprous halides, CuI, CuBr, and CuCl, were obtained from commercial sources and the material was used directly from the bottle. The impurity content of these compounds as observed under the microscope was estimated to be less than 1 percent by volume. The stable form of the cuprous halides at room pressure and temperature is the sphalerite or zinc blende structure in which the metal atoms are surrounded by four halogen atoms [11]. Under pressure the cuprous halides undergo phase transformations with differences in absorptions in the visible spectrum. The only previously reported cuprous halide transformation was that for CuI given by Bridgman [12]. He found a transition at 14,000 bars based on measurements obtained from shearing and volume changes. Since the cuprous halides are similar structurally it was believed that their polymorphic forms would also show similarities.

## Cuprous Iodide

**Figure 5:**
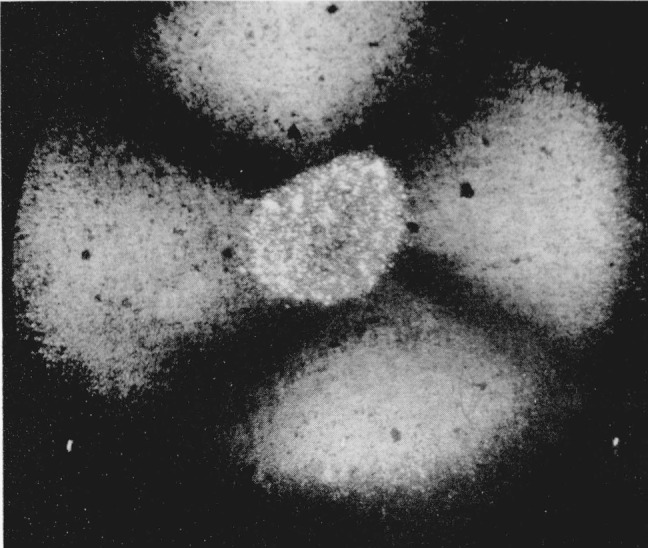
Cuprous iodide showing birefringent phase between crossed nicols at 4,000 bars. Magnification 160×

**Figure 6:**
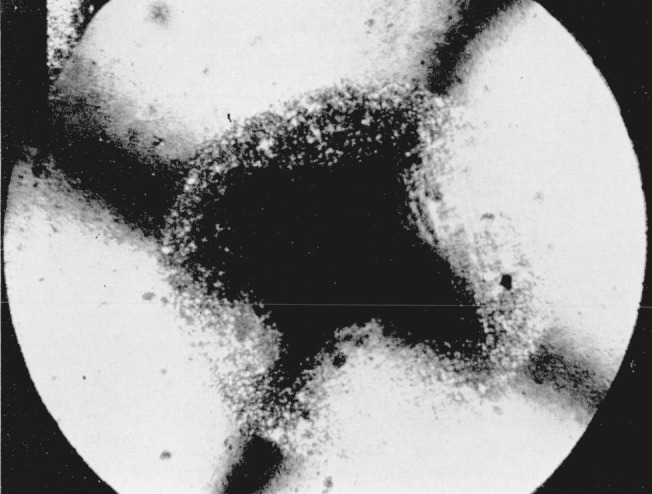
Cuprous iodide showing outer birefringent phase with central cubic phase. Under crossed nicols at a pressure of 5,000 bars. Magnification 400×.

**Figure 7:**
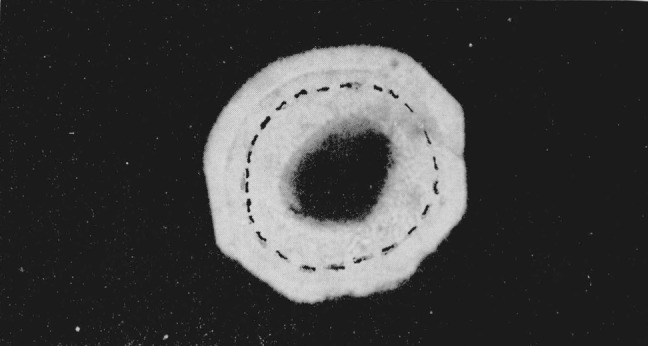
Cuprous iodide showing three phases. Pressure 40 kilobars. Magnification 160×.

Cuprous iodide appears to have three transitions. At a calculated pressure of 4,000 bars the sphalerite form of cuprous iodide undergoes a transition to a phase which is optically birefringent. (See central area fig. 5 taken between crossed nicols.) The individual crystals have an average diameter of about 3 *μ* and their mean refractive index is estimated to be 0.008 higher than the zinc blende phase. The outer light areas segmented by the dark cross are the result of pressure birefringence in the diamonds as observed between nicols. As the pressure is increased by 1,000 bars the birefringent center moves outward and is replaced by a dark non birefringent center that suggest a phase transformation to a cubic modification (fig. 6). The refractive index difference between the two regions is small, estimated to be about 0.004, and it was difficult to see a Becke line. At a calculated pressure of approximately 15 kilobars a third transition takes place with the emergence of a phase with weak birefringence. This transition is believed to correspond to the transition reported by Bridgman. Figure 7 shows 3 transitions photographed in white light between uncrossed nicols. The boundary separating the two phases in the first transition can readily be seen but the boundary separating the two phases in the second transition has been sketched in by a dashed line. The third transition boundary is also readily visible and it will be noted that there is a lighter ring surrounding a dark inner area. The lighter ring contains the weakly birefringent phase and the inner dark area shows no birefringence. This area may represent another phase but the strong absorption prevents further identification. On releasing the pressure the transformations are reversible. However, the dark central area remains absorbing for several hours before returning to its normal transparency.

## Cuprous Bromide

**Figure 8:**
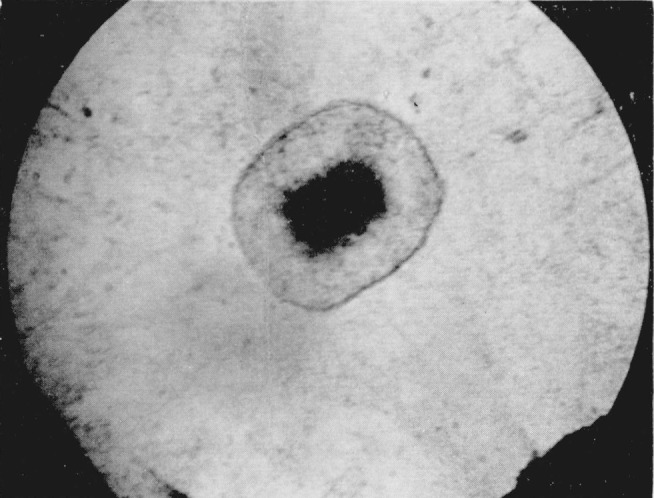
Cuprous bromide showing emergence of initial phase and dark center. Pressure 50 kilobars. Magnification 400×.

**Figure 9:**
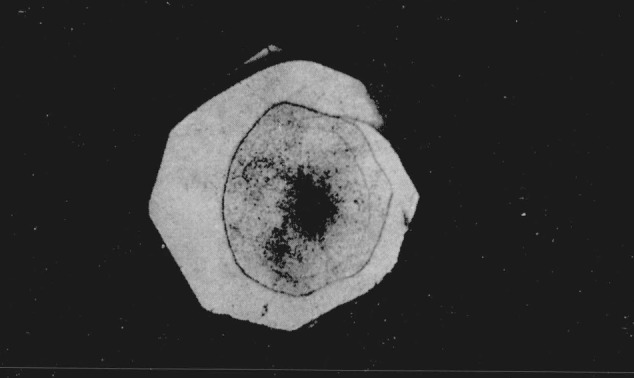
Cuprous bromide shows the two transitions with the absorbing center. Pressure 90 kilobars. Magnification 160×.

**Figure 10:**
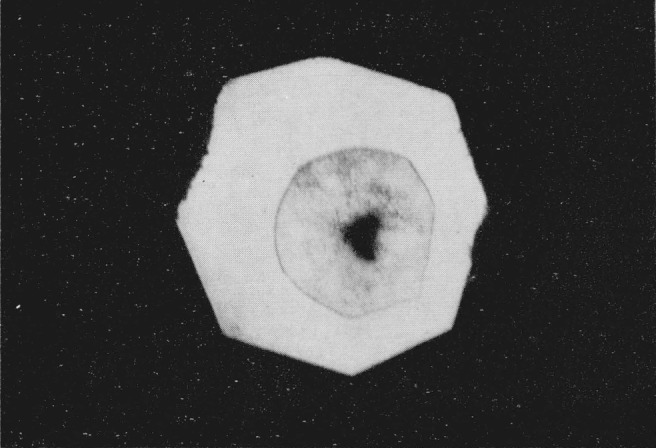
Conditions as in Fig. 8, but photographed at a wavelength of 505μ

**Figure 11:**
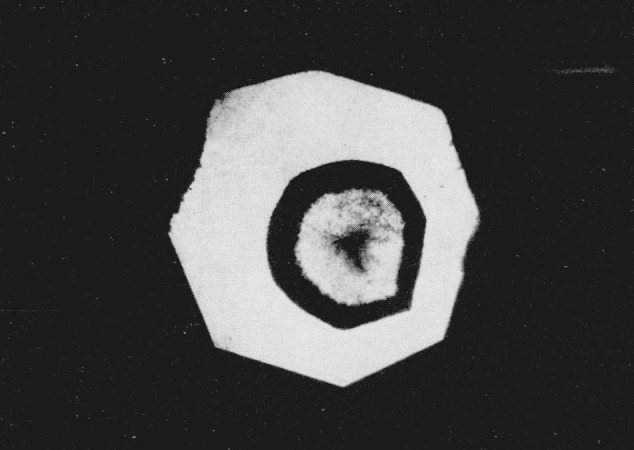
Conditions as in figure 8, but photographed at a wavelength of 475 μ

Cuprous bromide has three transitions, the first two occurring close to a calculated pressure of 47 kilobars, and the third occurring at a calculated pressure of 80 kilobars. Figure 8 taken at a calculated pressure of 50 kilobars shows the first and second transitions along with the darkening of the center. Phase number one is the white outer area of the photograph. Phase number two is shown by a darkened narrow zone line approximate 1 mm in width; phase number three a lighter region that has an amber color when viewed in white light. The dark central area does not appear to be a phase since it lacks a distinct boundary indicating a difference in refractive indices. This darkening is believed to be an absorption that occurs at all wavelengths in the visible spectrum. It was also observed that the absorption began just before the 47 kilobar transition when pressure was applied slowly. If pressure is applied quickly to the sample, going through the 47 kilobar transition to pressures of 60 kilobars or more, the central area does not develop an absorption area. As the zinc blende or original phase undergoes a transition at 47 kilobars one observes between crossed nicols the emergence of two phases at the same time; one is birefringent and the second is cubic and colored amber. The crystals of the birefringent phase, form the thin outer band as observed in figure 8. The manner in which these crystals develop and form a thin line suggests that they are stable only in a very narrow pressure range of 100 bars or less. In monochromatic light the amber phase shows a strong absorption beginning at 520 m*μ* and a total absorption at 475 m*μ.* The third transition was evidenced by the appearance of a light area (phase number four) that appeared to be faintly birefringent. The Becke line movement between the amber and weakly birefringent phase was slow, indicating the relative refractive indices were small, with an estimated difference of 0.008. There was no apparent absorption of the light phase in the visible range using monochromatic light. Figure 9 shows the occurrences of the four phases. Figure 10 is a photograph of CuBr taken in monochromatic light at a wavelength of 505 m*μ* and at a pressure of approximately 90 kilobars. At this wavelength the four phases show very little absorption. In comparison figure 11 is a photograph of the same area taken at 475 m*μ.* Phase number three and possibly number two are totally absorbing at this wavelength while phase number four is transparent.

When pressure is released the transitions are reversible. The darkened central area, however, remains for several hours before it becomes transparent.

## Cuprous Chloride

**Figure 12:**
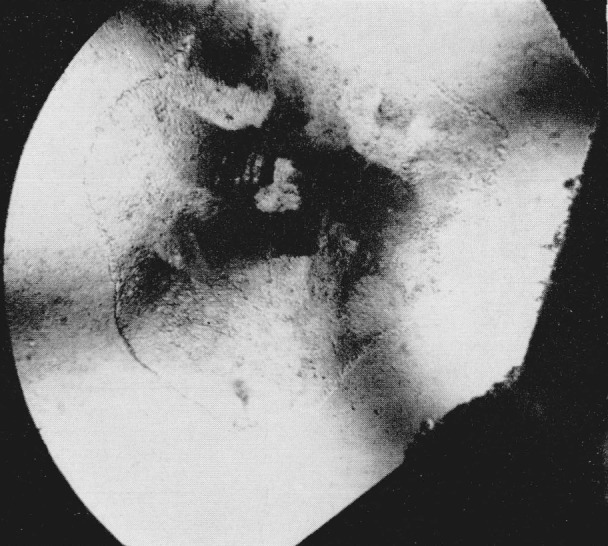
Cuprous chloride showing birefringent character of crystals under crossed nicols. Pressure 45 kilobars. Magnification 400×.

**Figure 13:**
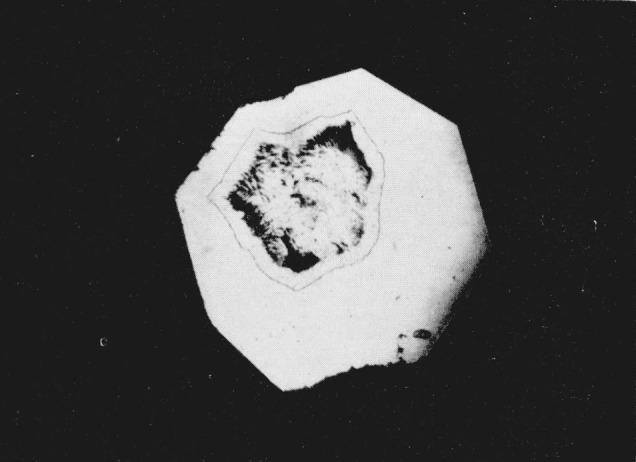
Cuprous chloride. Dark opaque areas in white transmitted light. Pressure 45 kilobars. Magnification 160 ×.

**Figure 14:**
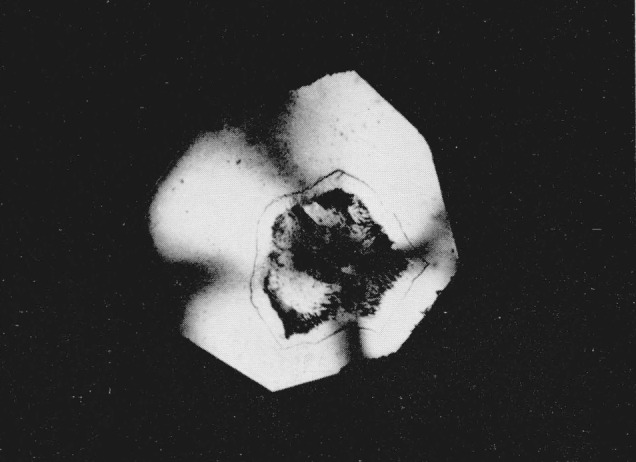
Cuprous chloride. Conditions as in figure 12, but photographed between crossed nicols.

**Figure 15:**
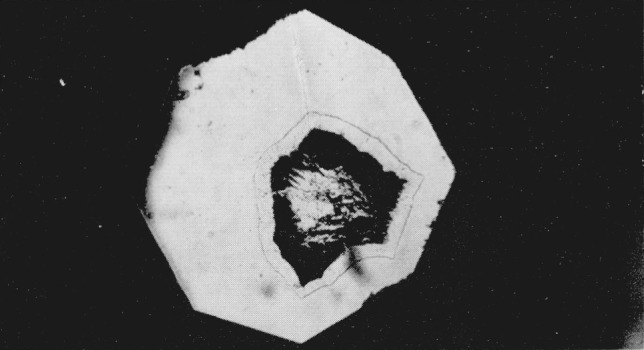
Cuprous chloride. Conditions as in figure 12, but photographed 4 days later.

**Figure 16:**
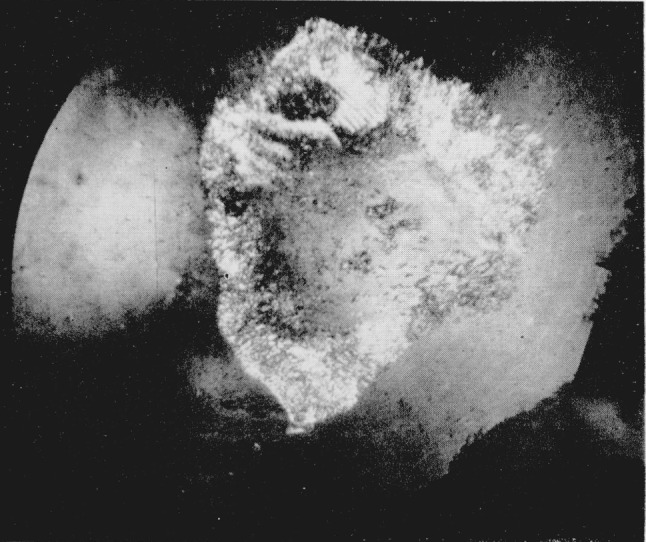
Cuprous chloride between crossed nicols showing birefringent areas formally occupied by opaque areas. Pressure 1 bar. Magnification 400 diam.

Two transformations were detected in cuprous chloride in the pressure range 0 to 100 kilobars. The first transition occurred at a calculated pressure of 42 kilobars, while a second occurred at approximately 55 kilobars. The second transition was very sluggish and it was almost missed. The initial zinc blende structure transformed to a weakly birefringent phase with crystals up to 30 *μ* in length. Between crossed nicols the crystals have an estimated birefringence of about 0.006 and their extinction positions were well-defined. See figure 12. The difference of the mean refractive index between the phases is estimated to be 0.015 as determined by the Becke line movement. The difference would give a volume change in the range of 10 to 15 percent. After a period of 12 hr or longer at a pressure of 60 kilobars an area within the birefringent phase became opaque when observed in white transmitted light. The outside of the darkened area showed a definite boundary but the inner portions were irregular. See figure 13. Figure 14 is the same area taken between crossed nicols and one can observe the birefringent crystals similar to those observed in figure 12. Figure 15 shows the same area taken with uncrossed nicols four days later. As compared with figure 13, it can be seen that the opaque area has greatly increased in size and is completely absorbing in the visible spectrum. As pressure is decreased from 60 kilobars to approximately 30 kilobars the opaque areas transform to a transparent brown color in white light. Between crossed nicols at one bar pressure, the area formally opaque at 60 kilobars becomes granularly birefringent as shown in figure 16. This phase has refractive indices slightly higher than the cubic zinc blende structure. After a period of 24 hr the birefringent phase transforms back to a cubic material.

## Summary of Cuprous Halides

In general the cuprous halides do not all have the same transition characteristics either with respect to their transition pressures or with respect to their transition phases. The initial transition of the cuprous halides is from a cubic zinc blende type structure to a birefringent phase. As is shown in table 2 these transformations do not all occur at the same pressure although the transition pressures of CuBr and CuCl are of the same order of magnitude. The pressures as given in table 2 are believed to be correct within a limit of 5 percent. In transition number 2 birefringent phases of CuI and CuBr appear to transform into a cubic phase whereas CuCl appears to transform into a second birefringent phase. The transformation of CuCl into an opaque phase (fig. 13) is sluggish. This phase is considered to be anisotropic since at lower pressures the material becomes transparent and birefringent. It is quite possible, however, that the opaque area is cubic in character, its optical transmission being masked by the total absorption in the visible spectrum.

## Figures and Tables

**Table 1:** 

Silver iodide		Silver bromide	Silver chloride
			
	N	N	N
High	12.430	2.442	2.239
Low sphalerite	2.232	2.429	2.221
			
Difference	0.198	0.013	0.018

1 The sodium chloride or amber colored phase.

**Table 2:** Transition pressures

	1	2	3
			
CuI	4,000 bars	5,000 bars	15,000 bars
CuBr	47,000 bars	47,000 bars	80,000 bars
CuCl	42,000 bars	55,000 bars	

## Footnotes

1 Figures in brackets indicate the literature references at the end of this paper.

2 The author wishes to support the recommendation of the informal committee on high pressure measurements at the Lake George International Conference on High Pressures, 1960 [3], that the bar be universally used as a standard pressure measurement in high pressure work.
